# Epigenetic regulation of inflammation in localized aggressive periodontitis

**DOI:** 10.1186/s13148-017-0385-8

**Published:** 2017-09-02

**Authors:** L. M. Shaddox, A. F. Mullersman, H. Huang, S. M. Wallet, T. Langaee, I. Aukhil

**Affiliations:** 1 Department of Periodontology, University of Florida College of Dentistry, P.O. Box 100434, Gainesville, FL 32610-0434 USA; 2 Department of Oral Biology, University of Florida College of Dentistry, Gainesville, FL USA; 3 Center for Pharmacogenomics, University of Florida, Gainesville, FL USA

**Keywords:** Inflammation, Aggressive periodontitis, Toll-like receptors, Epigenetics, Leukotoxins

## Abstract

**Background:**

We have previously demonstrated a Toll-like receptor (TLR)-mediated hyper-responsive phenotype in our cohort of localized aggressive periodontitis (LAP) individuals. However, mechanisms related to this phenotype are still not clear in the literature. The objective of this cross-sectional study is to examine the role of epigenetic regulation, specifically DNA methylation status of genes in the TLR pathway in this cohort. Peripheral blood was collected from 20 LAP patients and 20 healthy unrelated controls. Whole blood was stimulated with 1 μl (100 ng/μl) of purified *Escherichia coli* lipopolysaccharide (LPS) for 24 h and cyto/chemokines in the supernatants analyzed by Luminex multiplex assays. Genomic DNA extracted from buffy coats prepared from a second tube of whole blood was used for DNA methylation analysis by pyrosequencing of seven TLR signaling genes (FADD, MAP3K7, MYD88, IL6R, PPARA, IRAK1BP1, RIPK2).

**Results:**

Significant differences in the methylation status were observed at specific CpG positions in LAP patients compared to healthy controls and interestingly also between severe and moderate LAP. Specifically, subjects with moderate LAP presented hypermethylation of both the upregulating (MAP3K7, MYD88, IL6R, and RIPK2) and downregulating (FADD, IRAK, and PPARA) genes, while severe LAP presented hypomethylation of these genes. Further analysis on CpG sites with significant differences in methylation status correlates with an increased pro-inflammatory cytokine profile for LAP patients.

**Conclusions:**

Our findings suggest that epigenetic modifications of genes in the TLR pathway may orchestrate the thresholds for balancing induction and prevention of tissue destruction during the course of disease, and thus differ significantly at different stages of the disease, where moderate LAP shows hypermethylation and severe LAP shows hypomethylation of several genes.

**Trial registration:**

https://clinicaltrials.gov, NCT01330719

## Background

Periodontal disease is one of the most prevalent chronic oral infectious/inflammatory diseases in the USA. It affects one in every two North Americans [1]. The etiology of inflammatory periodontal diseases involves a multitude of factors, both intrinsic and extrinsic, that together determine clinical presentation. This disease is first initiated from a previous infection of the gingival tissues, mainly of Gram-negative bacteria, which elicits a host immune response that plays a major role in the progression and severity of the disease. The inflammatory response plays both a beneficial and harmful role in the pathogenesis of periodontitis, functioning to remove bacterial infection but concomitantly harming surrounding tissues [2]. Although of less frequent nature than the chronic forms of the disease, the abnormal immune response seen in localized aggressive periodontitis (LAP), known as a “hyper-responsive” phenotype, may result in the aggressive and rapid connective tissue loss and alveolar bone resorption that may lead to early tooth loose in young individuals [3]. While it is understood that bacteria may be an etiological agent in periodontal disease, susceptibility to inflammation has been shown to be influenced by intrinsic factors, such as genetics. Interestingly, LAP is known to be associated with familial aggregation [4–8]. Previous research from our group has shown that LAP individuals, and to a lesser extent their unaffected siblings, exhibit an elevated immune response when compared to healthy controls, further suggesting that genetics may play a role in this hyper-responsiveness [3]. Recent research using genome-wide analysis has found that differences in genes related to the inflammatory response are associated with an increased risk of periodontal disease [9]. In addition, it has been shown that a portion of these inflammatory genes may be regulated by epigenetic modifications [10].

Epigenetics is the study of heritable changes in gene expression that are not caused by changes in the DNA sequence, which is distinctly different from genetic variations in the population, such as single nucleotide polymorphisms [11]. Epigenetic regulation has become an important topic in medical research, and current literature has linked these changes to certain cancers, cardiovascular, and autoimmune diseases [12]. Through pyrosequencing, the methylation of a cytosine base within a CpG dinucleotide in the promoter region of an inflammatory gene can be accurately measured and used to determine the association with inflammatory response or phenotype. In most cases, hypermethylation of promoter DNA sequences is highly correlated with gene silencing, while DNA hypomethylation correlates with increased gene expression [9]. While limited research has been dedicated to epigenetics in periodontal disease, recent studies have linked the DNA methylation status in the promoter regions of various inflammatory genes to periodontitis [10, 13–15] and to cytokine expression in diseased periodontal tissue [12]. Thus, recent evidence suggests a role of epigenetic modifications in the inflammatory pathway of periodontitis. However, further studies are needed to evaluate this role in specific localized and aggressive forms of periodontitis.

Toll-like receptors (TLRs) are important single, membrane-spanning, non-catalytic receptors that recognize lipopolysaccharides from bacteria resulting in activation of signaling cascade culminating in the activation of many inflammation-associated genes. Signaling molecules in the TLR pathways can be pro- or anti-inflammatory. Expression and activation of these signaling molecules can result in either upregulation (myeloid differentiation primary response 88 (MYD88), mitogen-activated protein kinase kinase kinase 7 (MAP3K7), receptor interacting serine/threonine kinase 2 (RIPK2), interleukin-6 receptor (IL6R)) or downregulation (Fas associated via death domain (FADD), peroxisome proliferator-activated receptor alpha (PPARA), interleukin-1 receptor-associated kinase 1 binding protein 1 (IRAK1BP1)) of TLR-mediated inflammation. MAP3K7, member of the serine/threonine protein kinase family, activates NF-*ƙ*B in the TLR signaling pathway [16], as well as RIPK2, a dual-specificity kinase that can associate with the TNF receptor (TNFR) leading to activation of NF-*ƙ*B and induction of apoptosis [17, 18]. MYD88 is a canonical cytoplasmic adapter protein involved in TLR signaling pathways, acts via IRAK1, IRAK2, and IRAK7 to regulate cytokine secretion, and has a more central role in the inflammatory pathways [19]. IL6R is a receptor for the pleiotropic cytokine IL-6 that regulates cell growth and differentiation as well as immune response, requires a second transmembrane protein gp130 as a signaling subunit, and forms a complex with IL-6-IL6R-gp130. IL6R can be proteolytically cleaved from the cell membrane generating a soluble IL6R (sIL6R) that can still bind to IL-6 [20]. Classical signaling via the membrane-bound IL6R is usually protective while the trans-signaling that occurs with sIL6R is pro-inflammatory. Regarding genes involved in downregulating inflammation, FADD is an adapter molecule that is recruited by tumor necrosis factor receptor (TNFR) and along with caspase 8, and participates in the death signaling initiated by TNFR (extrinsic pathway) [21]. FADD and caspase 8 act as pro-survival factors by promoting the cleavage and inactivation of RIPK1 and RIPK3, thereby suppressing the deleterious effects of necrosis [21]. PPARA has anti-inflammatory effects and inhibits NF-*ƙ*B signaling pathway by inducing IƙBα leading to decreased expression of pro-inflammatory cytokines [22]. IRAK1BP1 is anti-inflammatory in functional nature and modulates the inflammatory response by influencing the relative ratios of endogenous NF-*ƙ*B subunits [23].

The objective of this study is to examine the role of DNA methylation status in the promoter regions of genes involved in TLR signaling pathways in LAP patients, potentially providing a link between methylation pattern and disease as well as the degree of inflammatory hyper-responsiveness. The few recent studies in this area focus on epigenetics solely in gingival tissues, whereas our focus is on overall inflammatory host response in aggressive periodontitis.

## Results

**Fig. 1:**
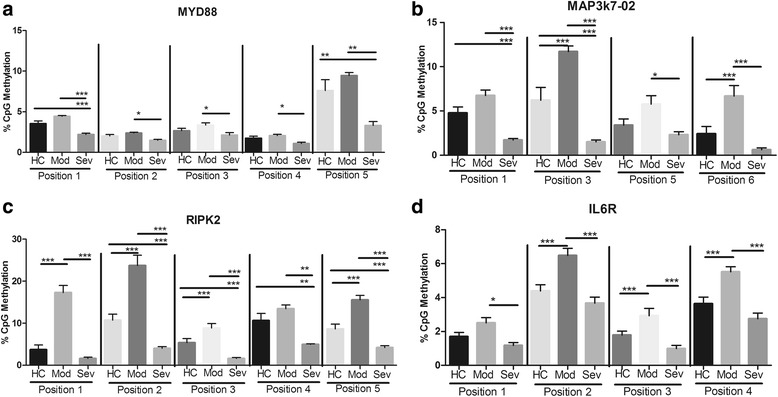
DNA methylation levels of pro-inflammatory genes in periodontitis vs control subjects. ANOVA with Tukey’s comparisons or Kruskal-Wallis with Dunn’s comparisons were carried out among healthy controls (*HC*), localized aggressive moderate (*Mod*), and severe (*Sev*) periodontitis. ***p < 0.001, **p < 0.01, *p < 0.05. Note that severe aggressive periodontitis displays lower methylation levels in several genes when compared to moderate periodontitis, while moderate disease shows higher methylation levels in general. TLR-2 signaling genes that are pro-inflammatory: *RIPK2* receptor-interacting serine/threonine-protein kinase 2(**c**), *MP3K7* mitogen-activated protein kinase kinase kinase 7(**b**), *MYD88* myeloid differentiation primary response gene 88(**a**), *IL6R* interleukin 6 receptor(**d**)

**Fig. 2:**
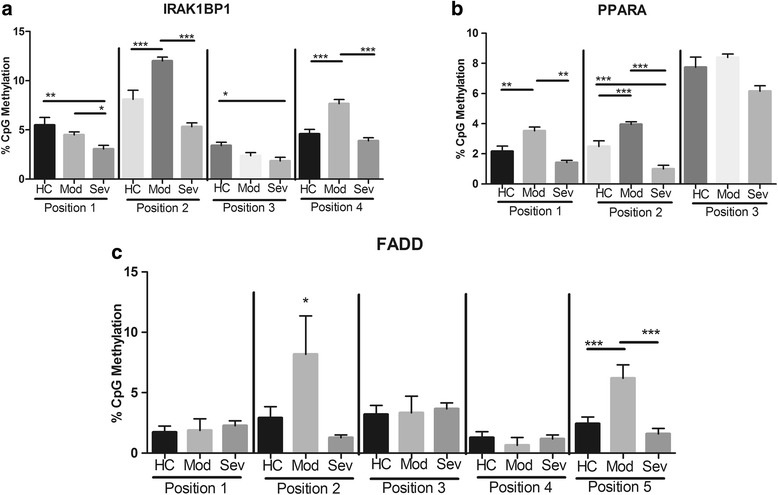
DNA methylation levels of anti-inflammatory genes in periodontitis vs control subjects. ANOVA with Tukey’s comparisons or Kruskal-Wallis with Dunn’s comparisons were carried out among healthy controls (*HC*), localized aggressive moderate (*Mod*), and severe (*Sev*) periodontitis. ***p < 0.001, **p < 0.01, *p < 0.05. Note that severe aggressive periodontitis displays lower methylation levels in several genes when compared to moderate periodontitis, while moderate disease shows higher methylation levels in general. TLR-2 signaling genes that are anti-inflammatory: *FADD* Fas-associated protein with death domain(**c**), *IRAKBP1* interleukin 1 receptor-associated kinase 1 binding protein 1(**a**), *PPARA* peroxisome proliferator-activated receptor alpha(**b**)

Table 1 shows demographic and clinical parameter information for all patients used in the analysis. The data on cytokine expression by peripheral blood cells in this population has been previously described [3]. Genomic DNA from peripheral blood leukocytes screened initially using the EpiTect Methyl II PCR Array Human Toll-Like Receptor Signaling Pathway Signature Panel from Qiagen showed seven genes involved in TLR signaling that showed differences in DNA methylation between healthy and diseased subjects (data not shown). Data on the selection parameters for DNA methylation analysis by bisulfite conversion and pyrosequencing for the seven genes are shown in Table 2. Data on the methylation status of the seven genes involved in TLR signaling are shown in Figs. 1 and 2. The DNA methylation status varied among the analyzed dinucleotide CpG sites, with several significant differences in methylation pattern in the promoter region for each of the seven genes involved in TLR signaling when comparing healthy controls, moderate LAP, and severe LAP patients. In general, there was a trend towards hypermethylation in moderate LAP patients, followed by a lowered methylation status for healthy controls and hypomethylation in severe LAP patients. This trend was observed in both the upregulators (MYD88, MAP3K7, RIPK2, IL6R) and downregulators (FADD, PPARA, IRAK1BP1) of the TLR pathway.

**Table 1:** Demographic and clinical parameters for all patients used in the analysis

	Age	Gender	PD (mm)	#PD > 6 mm	CAL (mm)	#CAL > 3 mm	% affected sites^a^	% BoP	% plaque
Severe LAP	13.70 ± 3.74	1M/9F	5.85 ± 0.9**	4.80 ± 2.4^#^	4.06 ± 1.24^#^	13 ± 10.49^#^	13.97 ± 8.54**	14.50 ± 9.54	45.60 ± 27
Moderate LAP	17.00 ± 2.21	5M/5F	5.38 ± 0.8**	1.60 ± 1.0	2.10 ± 0.45**	1.90 ± 1.14	8.30 ± 6.18**	14.20 ± 9.39	36.40 ± 17.85
HC	14.53 ± 5.39	8M/12F	2.74 ± 0.6	0	0	0	0	13.11 ± 9.44	22.84 ± 11.31

Means ± standard deviation. No statistical difference found between groups for gender, age, and BoP. *Note*: CAL value = 0 for healthy controls, as there is no attachment loss present in any site

*LAP* localized aggressive periodontitis, *HC* healthy controls/mild gingivitis (< 25% BoP and no radiographic bone loss or attachment loss), *PD* mean pocket depth of affected sites^a^ (PD ≥ 5 mm with CAL ≥ 2 mm and radiographic bone loss), *CAL* mean clinical attachment level of affected sites, *BoP* bleeding on probing, *NA* not applicable as healthy controls did not present sites PD ≥ 5 with CAL ≥ 2 mm

**p < 0.05 between LAP and HC; ^#^ *p* < 0.05 between severe vs moderate LAP and HC

**Table 2:**
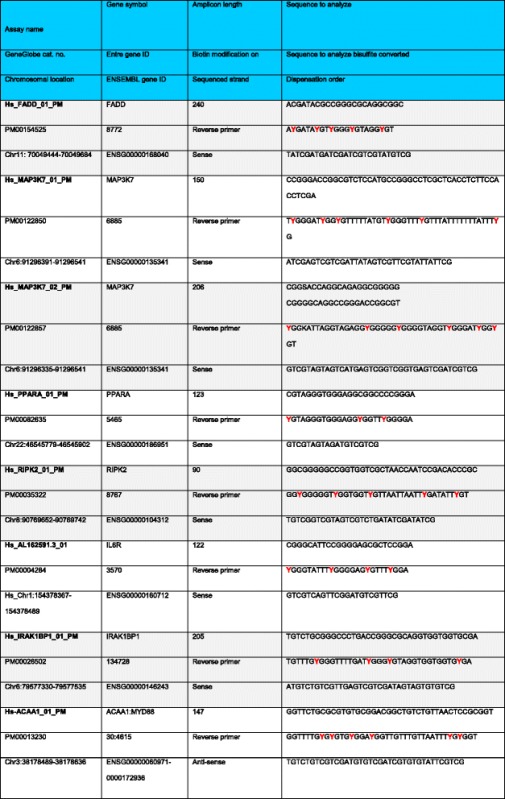
Data on gene symbols, chromosome location, amplicon length, and potential methylation sites for genes in the TLR signaling pathway

Potential methylation sites in red/italics for seven genes in the TLR signaling pathway used in the detailed analysis following an initial screening using the EpiTect Methyl II PCR Array

### Upregulators of TLR pathway (MYD88, MAP3K7, RIPK2, IL6R)

The methylation status in the upregulators of the TLR pathway showed significant differences among the groups for all but one site measured (Fig. 1). MAP3K7 has a large promoter region, and in order to complete our analysis, two primers were utilized. MAP3K7-01 showed increased methylation in moderate LAP compared to severe LAP at position 1 (*p* < 0.01), position 2 (*p* < 0.01), position 3 (*p* < 0.05), position 4 (*p* < 0.001), and position 6 (*p* < 0.001). Additionally, MAP3K7-01 position 4 and position 5 indicated hypermethylation of moderate LAP when compared to both severe LAP and healthy controls (*p* < 0.001). MAP3K7-01 position 5 did not show any significant differences in methylation status, but there was a trend towards hypermethylation in moderate LAP patients. MAP3K7-02 had similar methylation patterns observed in MAP3K7-01, with increased methylation levels in moderate LAP compared to severe LAP at position 1 (*p* < 0.001), position 3 (*p* < 0.001), position 5 (*p* < 0.05), and position 6 (*p* < 0.001). Comparing the methylation status to healthy controls, moderate LAP showed significantly higher methylation levels at position 3 and position 6 (*p* < 0.001). In a similar comparison of the methylation status to healthy controls, severe LAP patients showed significantly lower levels at positions 1 and 3 (*p* < 0.001).

RIPK2 showed highly significant differences in methylation status among groups for all CpG sites measured. Comparing the methylation between healthy controls and moderate LAP, there was an increased amount of methylation in moderate LAP patients at positions 1, 2, 3, and 5 (*p* < 0.001). Similarly, there was a significantly increased amount of methylation in moderate LAP when compared to severe LAP at all five positions measured: positions 1, 2, 3, 5 (*p* < 0.001) and position 4 (*p* < 0.01). A comparison of healthy controls to severe LAP shows hypomethylation of severe LAP patients at positions 2, 3, 5 (*p* < 0.001) and 4 (*p* < 0.01). MYD88 showed consistently higher methylation in moderate LAP when compared to severe LAP at all sites: positions 1 (*p* < 0.001), 2, 3, and 4 (*p* < 0.05), and 5 (*p* < 0.01). Two sites on the MYD88 promoter region showed higher levels of methylation in moderate LAP as compared to severe LAP, at positions 1 (*p* < 0.001) and 5 (*p* < 0.01). In addition, two sites showed lower methylation in severe LAP as compared to healthy controls at positions 1 (*p* < 0.001) and 5 (*p* < 0.01). In a comparison of healthy controls and moderate LAP patients, there were no significant differences; only a trend for higher methylation in moderate LAP was observed.

The IL6R promoter region was also examined, and all four CpG sites measured showed significantly increased levels of methylation in moderate LAP patients. A comparison to healthy controls showed that moderate LAP presented increased methylation at positions 2 (*p* < 0.001), 3 (*p* < 0.001), and 4 (*p* < 0.001). Additionally, increased levels of methylation in moderate LAP were found when compared to severe LAP at positions 1 (*p* < 0.05), 2 (*p* < 0.001), 3 (*p* < 0.001), and 4 (*p* < 0.001).

### Downregulators of TLR pathway (FADD, PPARA, IRAK1BP1)

The downregulating genes analyzed on the TLR pathway provided several sites with statistically significant differences among patient groups (Fig. 2). FADD position 5 showed increased methylation in moderate LAP compared to healthy control (*p* < 0.001) and compared to severe LAP (*p* < 0.001). Position 2, although not significantly different, showed a similar trend. The additional FADD sites analyzed did not appear to have any trends in methylation patterns. The downregulator PPARA had increased methylation in moderate LAP compared to healthy controls at positions 1 (*p* < 0.01) and 2 (*p* < 0.001), as well as higher moderate LAP methylation when compared to severe LAP at positions 1 (*p* < 0.01) and 2 (*p* < 0.001). In addition, at position 2, severe LAP also showed lowered amounts of methylation as compared to healthy controls (*p* < 0.001).

IRAK1BP1 positions 2 and 4 followed a similar trend of increased methylation in moderate LAP as compared to both healthy controls and severe LAP (*p* < 0.001). Similarly, moderate LAP also showed higher levels of methylation vs severe LAP at position 1 (*p* < 0.05). Compared to healthy controls, severe LAP showed lower methylation at positions 1 (*p* < 0.01) and 3 (*p* < 0.05).

### Correlations with inflammatory markers

Significant correlations between methylation levels and cyto/chemokine-stimulated levels are shown in Table 3. Here, we noted significant positive correlations of FADD positions 2 and 5 and pro-inflammatory (GM-CSF, INFγ, IL1β, IL6, TNFα, among others) and the anti-inflammatory cytokine (IL10). On the other hand, methylation levels of MYD88 in all positions and IL6R position 3 showed significant negative correlations with several of these cyto/chemokines (Table 3).

**Table 3:**
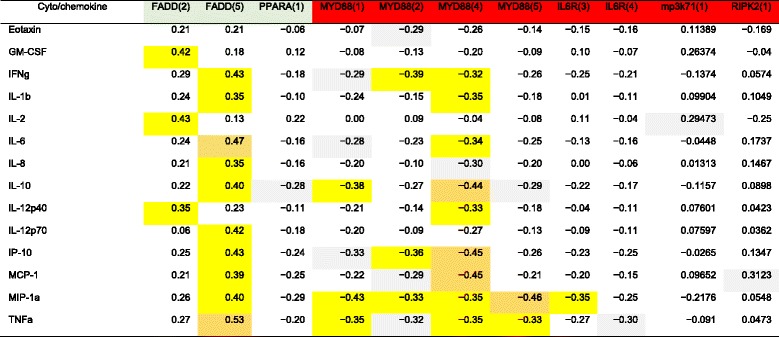
Correlations of different gene methylation and cyto/chemokine levels

Significant Spearman correlations (*r* value) of gene methylation and cyto/chemokine levels. Negative values indicate negative correlations. Gray boxes indicate *p* value > 0.05 but < 0.1, yellow boxes indicate *p* < 0.05, and orange boxes indicate *p* < 0.01

## Discussion

DNA methylation is the covalent methylation of the C5 position of cytosine residues in CpG dinucleotides, which leads to transcriptional repression of genes [24]. DNA methylation, typically associated with gene silencing, along with post-translational modifications of histone tails (associated with gene activation or silencing), are the most common mechanisms affecting accessibility of DNA to and binding of transcription factors. Thus, it is important to examine these epigenetic mechanisms to understand the mechanisms underlying chronic inflammatory conditions. The exact cause of the hyper-responsive inflammatory response observed in LAP patients [3] is unknown; however, our data here suggests that methylation status in specific genes may play a role in this disease and possibly in TLR-mediated signaling as different methylation patterns were found in disease and significant correlations were found between levels of methylation and lipopolysaccharide (LPS)-stimulated inflammatory cytokines. In addition to epigenetic factors, there may be other contributing factors to such an inflammatory response, such as differences in antibody response, specifically in IgG2 levels [25], and markedly so in black individuals [26]. It has also been demonstrated that this IgG response is influenced by certain cytokines, such as IL6, INFγ [27], and IL1 [28]. However, a few recent studies have also found a failure to mount a significant antibody response to other specific bacteria in LAP [29] and an autoimmune response to post-translationally modified self-antigens [30], which could also be contributing to a hyper-inflammatory profile. However, these factors were not evaluated in this study.

Previous studies have focused on analyzing DNA methylation of inflammatory cytokines and TLR2 genes from gingival biopsies [10, 13, 31–33]. Since we are interested in analyzing predisposition to LAP as it relates to the hyper-inflammatory response seen systemically in these patients, we examined methylation at specific CpG sites on the promoter regions of DNA from peripheral leukocytes. TLR signaling pathways are important in regulating the outcome of inflammatory diseases, and therefore, we have analyzed the methylation for some TLR signaling pathway genes, which results in either upregulation (MYD88, MAP3K7, RIPK2, IL6R) or downregulation (FADD, PPARA, IRAK1BP1) of TLR-mediated inflammation. It is interesting to note that overall, subjects with moderate severity of LAP had hypermethylation of both the upregulating and downregulating genes suggesting some kind of perpetual efforts by signaling molecules to orchestrate the thresholds for limiting, or preventing further, tissue destruction. In general, there was a trend towards hypomethylation of upregulating genes in severe LAP patients when compared to healthy controls and subjects with moderate disease severity. Some of the factors that influence DNA methylation include oxidative stress [34], smoking [35], infections [36], and dietary deficiencies in vitamin B, folate, methionine, choline, and zinc [37, 38]. More recently, microRNAs have also been suggested as regulators of DNA methylation [39]. Since inflammation also plays an important role in the modulation of DNA methylation patterns, it is often not clear as to the actual initiator of these aberrations in periodontal disease. A more likely scenario appears to be a linked feedback mechanism in which a specific methylation profile could be affecting the expression of genes involved in TLR signaling and the resultant overexpression of pro-inflammatory cytokines could be further attenuating the epigenetic control.

Among the pro-inflammatory TLR signaling molecules, nine of the 12 methylation sites in MAP3K7 showed significant hypomethylation in severe LAP subjects compared to either healthy subjects or those with moderate severity of disease. RIPK2, IL6R, and MYD88 all showed significant hypomethylation in subjects with severe LAP. Interestingly, subjects with moderate levels of LAP showed the most hypermethylation, quite often higher than even the healthy controls. It is tempting to speculate that once some initial stages of the disease set in, there may be some attempts at subduing inflammation by epigenetically downregulating expression of pro-inflammatory genes. A concomitant downregulation of anti-inflammatory genes, perhaps due to non-specific effects of some DNA methyltransferases could be keeping the disease status in a floating stage. This could explain why some subjects with mild to moderate levels of disease do not progress. The hypermethylation of both pro- and anti-inflammatory molecules of TLR signaling pathways observed in subjects with moderate LAP compared to healthy and severe LAP subjects can also be explained by other possibilities, such as variation in constituent leukocytes from buffy coats used to harvest gDNA [40] and possible change of methylation profiles during the progression of the disease, whereas at severe and progressive stages of disease, demethylation activity may occur, such as shown in cancer progression [41]. Thus, it is important and interesting to examine the role of different cell lines in peripheral blood responsible for these modifications and also to longitudinally test these individuals during the natural course of disease breakdown, although the latter is almost impossible, given LAP’s progressive nature and ethical aspects, and thus a limitation of the present investigation.

All three signaling molecules that presumably play a role in downregulating inflammation (FADD, PPARA, and IRAK1BP1) were also significantly hypomethylated in subjects with severe LAP compared to healthy controls and even subjects with moderate LAP. It appears that immune cells of subjects with severe LAP are recognizing the inflammatory events and are overexpressing the necessary molecules to suppress the pro-inflammatory cascade. The hypermethylation of FADD, PPARA, and IRAK1BP1 genes in subjects with moderate levels of disease could be one reason why the disease actually progresses slowly in these individuals. Interestingly, these hypotheses are actually corroborated by the fact that methylation levels of anti-inflammatory genes, such as FADD, correlated positively with both pro- and anti-inflammatory markers (such as INFγ, TNFα, and IL1β). In addition, the negative correlations of methylation levels of pro-inflammatory genes, such as MYD88, in several positions, with pro-inflammatory cytokines (such as INFγ, IL12p40 IP-10, MCP1, and MIP1α) also add to the hypothesis that the lower methylation of pro-inflammatory genes could lead to the expression of higher levels of these cytokines [19], and thus may corroborate with the aggressive tissue destruction seen in this disease, especially at severe stages. These are indeed only speculations at this point as we try to unveil these mechanisms and the role of epigenetics in LAP. Future studies should correlate these events with actual expression of these genes and then the resulting cytokine profile, which we intend to do in the near future. In addition, studies should develop DNA methylation profiles that would be specific for different stages of the disease and correlate this profile to the history of active or quiescent breakdown by carefully monitoring clinical signs of the disease course or even as it relates to treatment response, which also may prove or disprove some of these speculations above.

## Conclusions

In conclusion, our preliminary findings here suggest that the epigenetic profile of LAP, and most importantly the different stages of LAP, is distinct from healthy controls and may play a role in the active destruction of this disease. The moderate stages of disease show an overall hypermethylation of both pro- and anti-inflammatory genes, whereas severe stages show hypomethylation of all genes, suggesting that different stages of the disease activate genes in different ways in the ultimate attempt to subdue its progression and dissemination. Therefore, further studies need to carefully investigate these gene modifications, correlating these with the different clinical scenarios and activity of this aggressive disease.

## Methods

### Population

This was a cross-sectional study of epigenetic modifications in participants with LAP and healthy unrelated controls. Participants were recruited from the Leon County Health Department in Tallahassee, Duval County Health Department, in Jacksonville, and at the University of Florida, Gainesville, Florida, from 2007 to 2014. Informed consent was obtained and samples collected under a protocol were approved by the University of Florida Institutional Review Board (IRB). Inclusion criteria included age range of 5 to 25 years old, African-American race, diagnosed with LAP [42], or being an age, sex, and race-matched individual considered periodontally healthy/marginal gingivitis (< 25% bop and no attachment loss or radiographic bone loss). Selection of patients and the clinical protocols are described elsewhere [3].

### Methylation analysis

Peripheral blood was collected from 20 LAP (10 subjects with moderate and 10 with severe disease, according to the AAP classification) [42] and 20 healthy age-matched unrelated participants. Whole genomic DNA was extracted from the separated buffy coat (Qiagen Inc., Valencia, CA). An initial screening of the genomic DNA for changes in methylation of genes involved in TLR signaling was carried out using the EpiTect Methyl II PCR Array Human Toll-Like Receptor Signaling Pathway Signature Panel from Qiagen. Based on the initial screening of genomic DNA methylation from these subjects, more refined DNA methylation analysis was carried out for seven specific genes: FADD, MAP3K7, MYD88, PPARA, IRAK1BP1, RIPK2, and IL6R. The genomic DNA was bisulfite converted, and subsequent polymerase chain reaction (PCR) amplification of the specified genes was performed (Qiagen Inc., Valencia, CA). Pyrosequencing analysis of the PCR amplified products for DNA methylation was done at the University of Florida Center for Pharmacogenomics. Table 2 shows data on gene symbols, chromosome location, amplicon length, and methylation sites for seven genes in the TLR signaling pathway.

An additional 1 ml of peripheral blood was stimulated with 1 μl (100 ng/μl) of purified *Escherichia coli* LPS for 24 h (Qiagen Inc., Valencia, CA). Cytokine levels of LPS-stimulated blood were determined using the Luminex multiplex assay (EMD Millipore Corporation, Billerica, MA), as described previously [3].

### Statistical analysis

Percent methylation means were computed for each gene per group and were compared among groups using one-way analysis of variance (ANOVA). ANOVA with Tukey’s multiple comparisons was used when data was normally distributed, and Kruskal-Wallis with Dunn’s multiple comparisons was used when data was not normally distributed. Spearman correlations were run between each gene methylation and cyto/chemokine levels. A *p* value less than 0.05 was considered significant. Gender differences between LAP and controls were verified by chi-square. Clinical parameter analysis between LAP and controls were performed by *T* test or Mann-Whitney tests and among the three groups (severe LAP, moderate LAP, and controls) by ANOVA/Kruskal-Wallis, as described above.

## Abbreviations

AAP: American Academy of Periodontology
BoP: Bleeding on probing
CAL: Clinical attachment level
DNA: Deoxyribonucleic acid
FADD: Fas associated via death domain
HC: Healthy unrelated controls
IL6R: Interleukin-6 receptor
IRAK1BP1: Interleukin-1 receptor-associated kinase 1 binding protein 1
LAP: Localized aggressive periodontitis
LPS: Lipopolysaccharide
MAP3K7: Mitogen-activated protein kinase kinase kinase 7
MYD88: Myeloid differentiation primary response 88
PD: Pocket depth
PPARA: Peroxisome proliferator-activated receptor alpha
RIPK2: Receptor interacting serine/threonine kinase 2
TLR: Toll-like receptor
